# The associations of income and Black-White racial segregation with HIV outcomes among adults aged ≥18 years—United States and Puerto Rico, 2019

**DOI:** 10.1371/journal.pone.0291304

**Published:** 2023-09-18

**Authors:** Zanetta Gant, André Dailey, Xiaohong Hu, Wei Song, Linda Beer, Shacara Johnson Lyons, Damian J. Denson, Anna Satcher Johnson

**Affiliations:** Division of HIV Prevention, National Center for HIV, Viral Hepatitis, STD, and TB Prevention, CDC, Atlanta, GA, United States of America; Brown University, UNITED STATES

## Abstract

**Objective(s):**

To examine associations between Index of Concentration at the Extremes (ICE) measures for economic and racial segregation and HIV outcomes in the United States (U.S.) and Puerto Rico.

**Methods:**

County-level HIV testing data from CDC’s National HIV Prevention Program Monitoring and Evaluation and census tract-level HIV diagnoses, linkage to HIV medical care, and viral suppression data from the National HIV Surveillance System were used. Three ICE measures of spatial polarization were obtained from the U.S. Census Bureau’s American Community Survey: ICEincome (income segregation), ICErace (Black-White racial segregation), and ICEincome+race (Black-White racialized economic segregation). Rate ratios (RRs) for HIV diagnoses and prevalence ratios (PRs) for HIV testing, linkage to care within 1 month of diagnosis, and viral suppression within 6 months of diagnosis were estimated with 95% confidence intervals (CIs) to examine changes across ICE quintiles using the most privileged communities (Quintile 5, Q5) as the reference group.

**Results:**

PRs and RRs showed a higher likelihood of testing and adverse HIV outcomes among persons residing in Q1 (least privileged) communities compared with Q5 (most privileged) across ICE measures. For HIV testing percentages and diagnosis rates, across quintiles, PRs and RRs were consistently greatest for ICErace. For linkage to care and viral suppression, PRs were consistently lower for ICEincome+race.

**Conclusions:**

We found that poor HIV outcomes and disparities were associated with income, racial, and economic segregation as measured by ICE. These ICE measures contribute to poor HIV outcomes and disparities by unfairly concentrating certain groups (i.e., Black persons) in highly segregated and deprived communities that experience a lack of access to quality, affordable health care. Expanded efforts are needed to address the social/economic barriers that impede access to HIV care among Black persons. Increased partnerships between government agencies and the private sector are needed to change policies that promote and sustain racial and income segregation.

## Introduction

HIV continues to disproportionately affect Black/African American (Black) persons in the United States (U.S). Research reveals that socioeconomic differences between races account for a substantial portion of the racial disparity in many health outcomes, including infant mortality, heart disease, and cancer [1, 2]. At the same time, adjusting for socioeconomic differences does not eliminate racial disparities for all health outcomes. In other words, there is an independent contribution of racial status to disparities in specific health outcomes. These residual health differences may be due to a history of racial discrimination and residential segregation, as manifestations of structural racism, which has been recognized as a primary cause of health disparities [3–5].

Structural racism is defined as the “totality of ways in which societies foster [racial] discrimination, via mutually reinforcing [inequitable] systems… (e.g., in housing, education, employment, earnings, benefits, credit, media, health care, criminal justice, etc.) that in turn reinforce discriminatory beliefs, values, and distribution of resources” [6, 7]. Structural racism is reflected in history, culture, and interconnected institutions and includes the most influential socioecological levels at which racism may affect racial and ethnic health inequities [8]. Structural mechanisms (e.g., residential segregation) do not require the actions or intent of individuals, as they are constantly reconstituting the conditions necessary to ensure their perpetuation [8–10]. Even if interpersonal discrimination were eliminated, racial inequities would likely remain unchanged due to the persistence of structural mechanisms such as residential segregation [11].

Residential segregation has been a central mechanism by which racial inequality has been created and reinforced in the U.S. and has limited the socioeconomic mobility of Black persons by determining access to educational and employment opportunities [12]; Black persons are more segregated than other U.S. racial/ethnic minority groups [13]. Segregation, racial and economic, is a neglected but enduring legacy of racism in the U.S. and is a factor that contributes to higher rates of HIV diagnoses and poor health outcomes among Black persons. It does this by isolating Black persons from access to important resources and affecting neighborhood quality, with populations residing in lower income and relatively more isolated areas being more vulnerable [13–16]. Black persons tend to reside in communities with the highest social vulnerability in the U.S. [13, 17, 18]. Understanding the role of community-level social and structural factors—such as racial and economic segregation—is necessary to address these racial inequities.

Using methods such as the Index of Concentration at the Extremes (ICE) to explore income and racial segregation as proxies for structural racism is necessary to understand and address HIV diagnosis and care inequities that effect certain groups [19, 20]. Unlike other methods that measure residential segregation, such as the Gini index and the index of dissimilarity, ICE measures the extent to which an area’s residents are concentrated into groups at the extremes of deprivation and privilege, also referred to as spatial social polarization [21]. The spatial social polarization component of ICE takes into account the direction and extremities of residential segregation, and simultaneously captures geographic racial and/or income segregation, which helps identify the social determinants that may shape these communities, including spatial and economic access to healthcare services [21]. The ability of ICE to simultaneously capture racial and/or income disparities is important as residential segregation is a multi-dimensional construct that benefits from a multi-dimensional measure such as ICE [21]. ICE has been used to validate the association between income and race segregation and multiple poor health outcomes—including adverse birth outcomes, COVID-19 incidence and death rates, cancers, and mortality [22].

Assessing the role of segregation in contributing to poor health outcomes can provide information to inform interventions to increase health equity by addressing the inequitable concentration of Black persons in U.S. areas of deprivation. This paper examines associations between ICE measures for racial and economic segregation and HIV outcomes—specifically HIV testing, HIV diagnoses, linkage to HIV medical care, and viral suppression.

## Materials and methods

Data were obtained from 3 sources: Centers for Disease Control and Prevention’s (CDC’s) National HIV Prevention Program Monitoring and Evaluation (NHM&E) and National HIV Surveillance System (NHSS), and from the U.S. Census Bureau’s American Community Survey 2015–2019 5-year estimates (ACS). Per federal guidelines, NHM&E and NHSS are determined a public health activity and not human subject research; therefore, our study did not require institutional review board review or approval.

### HIV testing data

County-level HIV testing data for the U.S. and Puerto Rico were submitted to CDC’s NHM&E by 60 CDC-funded state and local health departments and 100 community-based organizations. Data included 2019 HIV testing data for adults aged ≥18 years that were linked to the ACS indicators using the 5-year estimates for 2015–2019 at the county level.

### HIV diagnoses, linkage to HIV medical care, and viral suppression data

Census tract-level data on HIV diagnoses, linkage to HIV medical care within 1 month of HIV diagnosis, and viral suppression within 6 months of HIV diagnosis were obtained from NHSS for adults aged ≥18 years with HIV diagnosed during 2019 in the U.S. and Puerto Rico. Linkage to care was measured by documentation of ≥1 CD4 or viral load (VL) tests ≤1 month of HIV diagnosis. A VL test result of <200 copies/mL indicates HIV viral suppression. VL test results were from the tests performed within 6 months of HIV diagnosis. Forty-five jurisdictions (44 states and the District of Columbia) submitted complete CD4 and viral load results to CDC to determine linkage to HIV medical care within 1 month and viral suppression within 6 months of HIV diagnosis. Data were not included for states and associated census tracts that do not have laws requiring reporting of all CD4 and viral load results or that had incomplete reporting of laboratory data to CDC. Areas without these laws were Idaho and New Jersey. Areas with incomplete reporting were Kansas, Kentucky, Pennsylvania, Vermont, and Puerto Rico.

Data included NHSS case data for adults aged ≥18 years with HIV diagnosed during 2019. Cases were geocoded to the U.S. census tract level based on residential address at the time of HIV diagnosis and linked to ACS indicators using the 5-year estimates for 2015–2019. Cases or census tracts were excluded if the address was nonresidential (e.g., military base, homeless shelter, corrections facility), a census tract could not be associated with the case, ACS information was not available for the census tract, or the assigned census tract could not be matched to a census tract provided by the ACS.

### ICE measures data

Three county-level and census tract-level ICE measures of spatial polarization were obtained from the 2015–2019 5-year ACS estimates: ICEincome, ICErace, and ICEincome+race.

We computed the ICE by using the following formula (21):

$$ICE_{i}=\frac{A_{i}-P_{i}}{T_{i}}$$

where

A_*i*_ = No. of privileged persons in county or census tract *i* (i.e., most privileged communities)

P_*i*_ = No. of deprived persons in county or census tract *i* (i.e., most deprived communities)

T_*i*_ = Total population with known information in county or census tract *i*

ICE ranges from -1, indicating 100% of the population is concentrated in the most deprived group to 1, indicating that 100% of the population is concentrated into the most privileged group. The ICE measures were categorized by quintiles, with Quintile 1 (Q1) representing the most deprived and Quintile 5 (Q5) representing the most privileged.

The 3 ICE measures were calculated as:

$$ICE_{{}_{\text{income}}}=\frac{No.\;of\;persons\;\geq \text{\$}100k_{i}\;-\;No.\;of\;persons\;<\text{\$}25k_{i}}{Total\;population_{i}}$$

referred to as income segregation, where positive values indicate counties or census tracts with larger concentrations of persons living in households with annual incomes ≥$100,000, and negative values indicate counties or census tracts with larger concentrations of persons living in households with annual incomes <$25,000.

$$ICE_{race}\;=\;\frac{\;No.\;of\;White\;persons_{i}\;-\;No.\;of\;Black\;persons_{i}\;}{Total\;population_{i}}$$

referred to as Black-White racial segregation, where positive values indicate counties or census tracts with larger concentrations of White residents, and negative values indicate counties or census tracts with larger concentrations of Black residents.

$$ICE_{income\;+\;race}\;=\;\frac{\;No.\;of\;White\;persons\;\geq \text{\$}100k_{i}\;-\;No.\;of\;Black\;persons\;<\text{\$}25k_{i}}{\;Total\;population_{i}}$$

referred to as Black-White racialized economic segregation, where positive values indicate counties or census tracts with larger concentrations of White residents living in households with annual incomes ≥$100,000, and negative values indicate counties or census tracts with larger concentrations of Black residents living in households with annual incomes <$25,000.

### Analysis

To assess the effects of income and racial segregation on the four HIV outcomes among adults aged ≥18 years, data were analyzed to determine differences in HIV outcomes by ICE quintiles. HIV diagnosis rates were calculated per 100,000 persons. The rate ratios (RRs) for HIV diagnoses and prevalence ratios (PRs) for testing, linkage, and viral suppression were estimated with 95% confidence intervals (CIs) to examine changes in HIV outcomes across ICE quintiles; Q5 (most privileged) was the reference group. The PR and RR 95% CIs that excluded 1 were considered statistically significant. Analyses were conducted using SAS software (version 9.4; SAS Institute).

## Results

Of 1,833,877 CDC-funded HIV tests administered to adults in 2019, most were among males assigned sex at birth (50.4%), aged 18‒34 years (57.7%), and Black/African American (39.9%) (Table 1). Across ICE measures, testing percentages were highest in Q2 for ICEincome (0.95%) and Q1 for ICErace (1.06%) and ICEincome+race (1.44%) (Table 2). Testing percentages were lowest in the most privileged (Q5) counties for all ICE measures (ICEincome = 0.58%, ICErace = 0.14%, and ICEincome+race = 0.43%). For all ICE measures, residing in Q1 through Q4 compared to the most privileged quintile (Q5) increased the likelihood of receiving a CDC-funded HIV test. Additionally, across all 3 ICE measures, the greatest PRs (i.e., higher likelihood in Q1 compared with Q5) for HIV testing were observed for ICErace (PR = 7.50; CI = 7.39–7.63) followed by ICEincome+race (PR = 3.38; CI = 3.36–3.39).

**Table 1:** Population characteristics for HIV outcomes and the Index of Concentration at the Extremes (ICE) values, 2019—United States and Puerto Rico.

	HIV Testing	HIV Diagnosis		Linkage to Care		Viral Suppression		
Variable	N	%	N	%	N	%	N	%
ICE								
ICE income^a^	1,833,877	—	28,371	—	21,250	—	18,178	—
ICE race^b^	1,833,877	—	28,919	—	21,634	—	18,524	—
ICE income and race^c^	1,833,877	—	28,369	—	21,251	—	18,178	—
Sex at Birth								
Male	924,240	50.40	24,172	80.88	18,156	81.25	15,513	81.12
Female	854,959	46.62	5,716	19.13	4,189	18.75	3,611	18.88
Missing	54,678	2.98						
Age								
18‒34	1,058,251	57.71	16,924	56.63	12,541	56.12	10,812	56.54
≥35	757,752	41.32	12,964	43.38	9,804	43.88	8,312	43.46
Invalid	17,874	0.97						
Race/Ethnicity								
American Indian/Alaska Native	11,269	0.61	134	0.45	107	0.48	90	0.47
Asian	34,641	1.89	540	1.81	435	1.95	396	2.07
Black/African American	731,938	39.91	13,037	43.62	9,611	43.01	8,131	42.52
Hispanic/Latino^d^	390,918	21.32	7,901	26.44	5,918	26.49	5,131	26.83
Native Hawaiian/other Pacific Islander	3,401	0.19	49	0.16	40	0.18	34	0.18
White	560,912	30.59	7,364	24.64	5,598	25.05	4,786	25.03
Multiracial	14,928	0.81	863	2.89	636	2.85	556	2.91
Missing/Unknown	85,870	4.68						
**Total**	**1,833,877**	**100**	**29,888**	**100**	**22,345**	**100**	**19,124**	**100**

^a^ICE income = (No. of persons ≥$100k − No. of persons <$25k) / total population.

^b^ICE race = (No. of White persons − No. of Black persons) / total population.

^c^ICE income and race = (No. of White persons with annual income ≥$100k − No. of Black persons with annual income <$25k) / total population.

^d^Hispanic/Latino persons can be of any race.

**Table 2:** HIV testing among adults aged ≥18 years, by Index of Concentration at the Extremes (ICE), 2019—county level^a^, United States and Puerto Rico.

HIV testing (ICE quintiles^f^)		ICE income^b^			ICE race (Black-White)^c^			ICE income and race (Black-White)^d^		
		Total tested	%^e^	PR (95% CI)	Total tested	%^e^	PR (95% CI)	Total tested	%^e^	PR (95% CI)
Q1 (most deprived)		112,146	0.87	1.49 (1.48–1.50)	1,029,830	1.06	7.50 (7.39–7.63)	506,952	1.44	3.38 (3.36–3.39)
Q2		211,512	0.95	1.62 (1.61–1.63)	580,517	0.70	4.92 (4.84–5.00)	445,008	0.75	1.77 (1.76–1.77)
Q3		253,869	0.78	1.34 (1.32–1.34)	163,175	0.38	2.69 (2.64–2.73)	272,803	0.76	1.77 (1.77–1.78)
Q4		478,563	0.90	1.54 (1.53–1.54)	45,004	0.23	1.61 (1.57–1.64)	290,905	0.59	1.39 (1.38–1.40)
Q5 (most privileged)		777,787	0.58	Reference	15,351	0.14	Reference	318,209	0.43	Reference
**ICE quintile cutpoints:**										
	**ICE_income:** median = –0.02; interquartile range (IQR) = –0.07 to 0.03; Q1 = –0.32 to –0.08; Q2 = –0.08 to –0.04; Q3 = –0.04 to –0.002; Q4 = –0.002 to 0.04; Q5 = 0.04 to 0.30									
	**ICE_race:** median = 0.81; IQR = 0.51 to 0.92; Q1 = –0.84 to 0.42; Q2 = 0.42 to 0.72; Q3 = 0.72 to 0.87; Q4 = 0.87 to 0.93; Q5 = 0.93 to 1.00									
	**ICE_income_race:** median = median = 0.08; IQR = 0.05 to 0.11; Q1 = –0.25 to 0.04; Q2 = 0.04 to 0.07; Q3 = 0.07 to 0.09; Q4 = 0.09 to 0.12; Q5 = 0.12 to 0.27									

**Abbreviations**: ICE = Index of Concentration at the Extremes; PR, prevalence ratio; Q1 = quintile 1; Q2 = quintile 2; Q3 = quintile 3; Q4 = quintile 4; Q5 = quintile 5; CDC, the Centers for Disease Control and Prevention [footnotes only].

**Note**. Data obtained from CDC’s National HIV Prevention Program Monitoring and Evaluation. Data were submitted by 60 CDC-funded state and local health departments and 100 community-based organizations to CDC.

^a^Data reflect the county of the person’s residential address at the time they received an HIV test.

^b^ICE income = (No. of persons ≥$100k − No. of persons <$25k) / total population.

^c^ICE race = (No. of White persons − No. of Black persons) / total population.

^d^ICE income and race = (No. of White persons with annual income ≥$100k − No. of Black persons with annual income <$25k) / total population.

^e^Row percent.

^f^ICE ranges from –1.0, indicating 100% of the population is concentrated in the most deprived group, to 1.0, indicating that 100% of the population is concentrated in the most privileged group. ICE measures were categorized into quintiles based on distribution among all U.S. counties from U.S. Census Bureau’s American Community Survey 2015‒2019 5-year estimates. PRs with 95% CIs were calculated to examine the relative difference across quintiles with Q5 (most privileged) counties; Q5 served as the reference group. Percentages were considered significantly different if the 95% CIs of the PRs excluded 1.0.

Among the 29,888 adults who received an HIV diagnosis in 2019, most were among males assigned sex at birth (80.9%), aged 18‒34 years (56.6%), and Black/African American (43.6%) (Table 1). Across ICE measures, diagnosis rates were highest in Q1 for all ICE measures (ICEincome = 22.5; ICErace = 28.2; ICEincome+race = 29.9) (Table 3), and lowest in Q5 for all ICE measures (ICEincome = 5.7; ICErace = 2.8; ICEincome+race = 4.9). For all ICE measures, the RRs (i.e., residing in Q1 through Q4 compared to the most privileged quintile [Q5]) increased the likelihood of receiving a diagnosis of HIV infection. Additionally, across all 3 ICE measures, the greatest RRs (i.e., higher likelihood in Q1 compared with Q5) for HIV diagnosis were observed for ICErace (RR = 9.93; CI = 9.39–10.50) followed by ICEincome+race (RR = 6.06; CI = 5.82–6.32).

**Table 3:** Diagnoses of HIV infection, linkage to HIV medical care within 1 month, and viral suppression within 6 months of HIV diagnosis among adults aged ≥18 years, by Index of Concentration at the Extremes (ICE)—United States and Puerto Rico (census tract level^a^), 2019.

HIV outcomes by ICE quintile^e^		ICE income^b^			ICE race (Black-White)^c^			ICE income and race (Black-White)^d^		
		Total	Rate	RR (95% CI)	Total	Rate	RR (95% CI)	Total	Rate	RR (95% CI)
**HIV diagnosis**										
Q1 (most deprived)		8,841	22.5	3.94 (3.78–4.10)	13,038	28.2	9.93 (9.39–10.50)	11,776	29.9	6.06 (5.82–6.32)
Q2		6,349	13.3	2.33 (2.23–2.43)	7,612	13.8	4.85 (4.58–5.14)	7,273	12.2	2.47 (2.36–2.57)
Q3		5,247	10.2	1.79 (1.71–1.87)	4,504	8.2	2.90 (2.73–3.08)	3,245	7.5	1.53 (1.45–1.60)
Q4		4,772	8.0	1.39 (1.33–1.46)	2,404	4.8	1.71 (1.60–1.82)	3,223	6.0	1.21 (1.15–1.28)
Q5 (most privileged)		3,162	5.7	Reference	1,361	2.8	Reference	2,852	4.9	Reference
Missing		1,518	—	—	969	—	—	1,518	—	—
**Linkage to care** ^**f**^										
Q1 (most deprived)		6,321	79.6	0.94 (0.92–0.96)	9,495	80.0	0.96 (0.93–0.98)	8,480	79.3	0.93 (0.91–0.95)
Q2		4,759	80.5	0.95 (0.93–0.97)	5,916	82.8	0.99 (0.96–1.02)	5,603	82.1	0.96 (0.94–0.98)
Q3		4,012	82.4	0.97 (0.95–0.99)	3,483	83.6	1.00 (0.97–1.03)	2,514	83.5	0.98 (0.96–1.00)
Q4		3,719	84.0	0.99 (0.97–1.01)	1,790	83.4	1.00 (0.97–1.03)	2,456	83.6	0.98 (0.96–1.00)
Q5 (most privileged)		2,439	84.5	Reference	950	83.6	Reference	2,198	85.3	Reference
Missing		1,094	81.7	—	711	77.6	—	1,094	81.7	—
**Viral suppression** ^**f**^										
Q1 (most deprived)		5,276	66.4	0.91 (0.89–0.94)	8,047	67.8	0.93 (0.89–0.96)	7,135	66.7	0.89 (0.86–0.91)
Q2		4,056	68.6	0.94 (0.92–0.97)	5,046	70.6	0.96 (0.93–1.00)	4,805	70.5	0.94 (0.91–0.96)
Q3		3,455	70.9	0.97 (0.95–1.00)	3,034	72.8	1.00 (0.96–1.04)	2,172	72.2	0.96 (0.93–0.99)
Q4		3,291	74.3	1.02 (0.99–1.05)	1,565	73.0	1.00 (0.95–1.04)	2,124	72.3	0.96 (0.93–0.99)
Q5 (most privileged)		2,100	72.8	Reference	832	73.2	Reference	1,942	75.3	Reference
Missing		946	70.6	—	600	65.5	—	946	70.6	—
**ICE quintile cutpoints:**										
	**ICE_income:** median = 0.03; interquartile range (IQR) = –0.07 to 0.13; Q1 = –1.00 to –0.09; Q2 = –0.09 to –0.01; Q3 = –0.01 to 0.06; Q4 = 0.06 to 0.16; Q5 = 0.16 to 1.00									
	**ICE_race:** median = 0.64; IQR = 0.23 to 0.86; Q1 = –1.00 to 0.11; Q2 = 0.11 to 0.51; Q3 = 0.51 to 0.75; Q4 = 0.75 to 0.89; Q5 = 0.89 to 1.00									
	**ICE_income_race:** median = 0.08; IQR = 0.01 to 0.15; Q1 = –1.00 to 0; Q2 = 0 to 0.06; Q3 = 0.06 to 0.10; Q4 = 0.10 to 0.16; Q5 = 0.16 to 1.00									

**Abbreviations**: CI = confidence interval; ICE = Index of Concentration at the Extremes; PR = prevalence ratio; Q1 = quintile 1; Q2 = quintile 2; Q3 = quintile 3; Q4 = quintile 4; Q5 = quintile 5; RR = rate ratio; VL = viral load; CDC, the Centers for Disease Control and Prevention [footnotes only].

**Note**. Data obtained from the National HIV Surveillance System. Data reflect the census tract of the person’s residential address at the time of receipt of an HIV diagnosis.

^a^Data reflect the census tract of the person’s residential address at the time of receipt of an HIV diagnosis. Rates = cases per 100,000 population

^b^ICE income = (No. of persons ≥$100k − No. of persons <$25k) / total population.

^c^ICE race = (No. of White persons − No. of Black persons) / total population.

^d^ICE income and race = (No. of White persons with annual income ≥$100k − No. of Black persons with annual income <$25k) / total population.

^e^ICE ranges from –1.0, indicating 100% of the population is concentrated in the most deprived group, to 1.0, indicating that 100% of the population is concentrated in the most privileged group. ICE measures were categorized into quintiles based on distribution among all U.S. census tracts from U.S. Census Bureau’s American Community Survey 2015‒2019 5-year estimates. RRs and PRs with 95% CIs were calculated to examine the relative difference across quintiles with Q5 (most privileged) census tracts; Q5 served as the reference group. Rates and percentages were considered significantly different if the 95% CIs of the RRs or PRs excluded 1.0.

^f^Linkage to care and viral suppression were assessed using data from 45 areas with complete reporting of lab data to CDC as of December 2021. Linkage to HIV medical care was measured by documentation of ≥1 CD4 or VL tests ≤1 month after HIV diagnosis. A VL test result of <200 copies/mL indicates HIV viral suppression. VL test results are within 6 months of diagnosis of HIV infection during 2019. Data not provided for states and associated census tracts that do not have laws requiring reporting of all CD4 and viral loads or that have incomplete reporting of laboratory data to CDC Areas without laws: Idaho and New Jersey. Areas with incomplete reporting: Kansas, Kentucky, Pennsylvania, Vermont, and Puerto Rico.

For linkage to HIV medical care within 1 month of diagnosis in 2019, the lowest percentages were in Q1 for all ICE measures (ICEincome = 79.6%; ICErace = 80.0%; ICEincome+race = 79.3%), and highest in Q5 for all ICE measures (ICEincome = 84.5%; ICErace = 83.6%; ICEincome+race = 85.3%) (Table 3). For viral suppression within 6 months of diagnosis in 2019, the lowest percentages were lowest in Q1 for all ICE measures (ICEincome = 66.4%; ICErace = 67.8%; ICEincome+race = 66.7%) and highest in Q4 for ICEincome (74.3%) and Q5 for ICErace = 73.2%) and ICEincome+race (75.3%) (Table 3). For all ICE measures where statistically significant differences were found, residing in Q1 through Q4 compared to Q5 decreased the likelihood of being linked to HIV medical care within 1 month of diagnosis or to have viral suppression within 6 months of diagnosis. Across all 3 ICE measures for linkage to care and viral suppression, the smallest PRs (i.e., lower likelihood in Q1 compared with Q5) were observed for ICEincome+race (linkage, PR = 0.93; CI = 0.91–0.95; viral suppression, PR = 0.89; CI = 0.86–0.91) followed by ICEincome (linkage, PR = 0.94; CI = 0.92–0.96; viral suppression, PR = 0.91; CI = 0.89–0.94).

## Discussion

This is the first large-scale county-level and census tract-level analysis to utilize ICE to assess the relationship between racial and economic segregation on HIV outcomes across the U.S and Puerto Rico. This analysis found that adults who resided in the most privileged communities (Q5) have substantially better HIV outcomes (i.e., diagnosis, linkage to care, viral suppression) than adults in the most deprived communities (Q1). For income, Black-White racial segregation, and Black-White racialized economic segregation, higher HIV testing percentages and diagnosis rates and lower linkage to HIV medical care and viral suppression percentages were observed in the most deprived compared with the most privileged communities. The highest PRs for HIV testing percentages and RRs for diagnosis rates were observed for ICErace, and lowest PRs for linkage to care and viral suppression were observed for ICEincome+race.

Our findings of higher percentages of HIV testing in more deprived communities were unexpected and did not align with the worst HIV outcomes (i.e., diagnosis, linkage to care, viral suppression). This can be explained by CDC-funded testing efforts being focused on high-priority populations, which might also partially explain the higher HIV diagnosis rates in these communities. However, social determinants of health (SDOH) factors shaped by income, education, wealth, and childhood and neighborhood socioeconomic conditions, which vary systematically by race/ethnicity groups, also explain higher diagnosis rates as well as our finding of lower percentages of linkage to care and viral suppression in these communities [14, 23, 24]. These findings suggest spatial social polarization, as demonstrated by the ICE measures, might contribute to poor HIV outcomes and disparities for Black adults by segregating them in more deprived communities [14].

For HIV testing percentages and diagnosis rates, across quintiles, PRs and RRs were consistently greatest for ICErace, where communities with the highest concentrations of Black residents had higher testing percentages and diagnosis rates than communities with the highest concentrations of White residents. For linkage to care and viral suppression, across quintiles, PRs were consistently lower for ICEincome+race, where communities with the highest concentrations of Black residents living in households with annual incomes <$25,000 had linkage and viral suppression percentages that were lower than those of communities with the highest concentrations of White residents living in households with annual incomes ≥$100,000. This is consistent with previous research that examined racial residential segregation [25]. Residential segregation remains pervasive and may influence health by concentrating poverty, environmental pollutants, infectious agents, and other adverse conditions [12, 26]. For instance, Morello-Frosch and Jesdale [27] found that segregation increased the risk of cancer related to air pollution. Studies using multilevel modeling that simultaneously accounts for individual and structural factors also find associations between segregation and illness [28, 29]. Our findings suggest that Black-White racial segregation and Black-White racialized economic segregation contribute to adverse health outcomes more than income segregation alone. In other words, Black-White racial segregation alone or in combination with economic segregation plays an important role in the production of inequitable and adverse health outcomes (e.g., lack of access to quality, affordable health care) by unfairly concentrating Black persons in highly segregated, deprived communities [14].

To our knowledge, this is the first time ICE has been used to analyze county-level and census tract-level data for HIV outcomes. The use of this novel measure provides evidence to support our hypothesis that the worst HIV outcomes (i.e., diagnosis, linkage to care, viral suppression) occur in the most deprived communities. This analysis also adds to the literature by quantifying the negative effects that structural racism (as measured by income, racial, and racialized economic segregation) has on HIV care and treatment outcomes when there is more Black-White racial segregation. These results can be used to inform policy and programmatic efforts that support investments in these communities and equitable redistribution of resources that improves the health of all persons.

This work further suggests that more action and innovative strategies are needed to achieve HIV diagnosis and treatment equity when there is increased Black-White segregation. For example, an innovative strategy might include use of implementation science from the Ryan White HIV/AIDS Program (RWHAP) Special Projects of National Significance Program (SPNS), which supports the development of pioneering HIV care and treatment models that could evaluate the design, implementation, utilization, cost, and health-related outcomes of treatment strategies that address disparities that are a result of Black-White segregation and other forms of structural racism [30]. In addition to SPNS and RWHAP-funded facilities, Federally Qualified Health Centers (FQHCs) serve as another avenue to improve the health of medically underserved populations including persons with HIV [31]. FQHCs aim to provide affordable, accessible, high-quality healthcare in areas where economic, geographic, or social barriers limit healthcare access to reduce health disparities [31]. Accelerated implementation of HIV testing strategies that include rapid linkage to care and treatment is needed to identify persons with infection to increase viral suppression or linkage to prevention efforts [32]. For example, the District of Columbia’s Red Carpet Entry Program is a structural-level intervention that used an improved, redesigned referral network to link 70% of persons with diagnosed HIV to care within 72 hours [33]. Expanded efforts should continue to address access to health care and social and economic barriers that might impede access [32].

In addition to efforts to ameliorate consequences of segregation, increased partnerships between government agencies and private sector are needed to change policies that promote and sustain segregation [34]. Exclusionary zoning (a form of structural racism that results in residential segregation) is a legal practice used for decades that keeps affordable housing out of neighborhoods through land use and building code requirements. This practice keeps lower-income persons―who are disproportionately racial minorities― out of wealthy and middle-class neighborhoods [35]. To address structural racism and segregation in neighborhoods, the U.S. Department of Housing and Urban Development (HUD) recently reinstated the Fair Housing Act’s Affirmatively Furthering Fair Housing (AFFH) requirement, which requires HUD and its funding recipients to address segregation and proactively take meaningful actions to overcome patterns of segregation, promote fair housing choice, eliminate disparities in opportunities, and foster inclusive communities free from discrimination [36].

Our analysis had several limitations that affect external validity. First, CDC-funded HIV testing efforts focus on high-priority populations who might reside in disadvantaged communities and might not represent national HIV testing patterns. Also, testing data were analyzed at the county-level and use of county as the unit of measurement may not fully account for the heterogeneity within them, as a smaller area may reflect the connection of social networks and physical spatial locations. Second, HIV diagnoses data might not be representative of all persons with HIV because not all persons with HIV have been tested or tested at a time when the infection could be detected and diagnosed. Third, linkage to care and viral suppression data were limited to 45 jurisdictions with complete reporting of laboratory data to CDC. These 45 jurisdictions represent 89% of all persons aged ≥13 years living with diagnosed HIV infection at year-end 2019 in the United States and are therefore not representative of data on all persons living with diagnosed HIV infection in the United States. Since CD4 and VL test results reported to HIV surveillance programs were needed to monitor the outcomes, not having these tests done or reported may prevent representation for all the outcomes in jurisdictions and monitoring of outcomes. Data on CD4 and VL test results during the follow‐up period may be delayed or missing for people who may have migrated to another jurisdiction (after HIV diagnosis) that did not report complete test results to CDC. Fourth, NHSS data were limited to people whose residential addresses were complete and could be geocoded (∼85.4%) and might not reflect the entire adult population with diagnosed HIV in those census tracts.

Our analysis also had limitations that affect internal validity. First, HIV outcomes vary by sociodemographic and environmental factors, such as age, sex, education, and income. Therefore, misclassification of outcome status and confounding may result in biased results. However, future analysis will incorporate multivariate analysis that includes sociodemographic variables such as age and sex. Second, not all persons with diagnosed HIV may have been linked to care within 1 month and/or virally suppressed within 6 months after diagnosis due to factors related to healthcare access, economic, geographic, and social barriers. These factors are unavailable and not monitored as part of routine HIV case surveillance, therefore, this study could not control for potential confounding due to them. Persons with diagnosed HIV who were rapidly linked to care and treatment may have less barriers to healthcare, resulting in better health outcomes, than persons who were not rapidly linked to care and treatment in the same community. Also, use of the first documented viral load (VL) test after HIV diagnosis to assess viral suppression within 6 months of diagnosis may not represent time to event but rather the opportunity for VL measurement, therefore, viral suppression within 6 months of diagnosis could be over- or underestimated.

## Conclusions

Understanding the impacts of structural racism on HIV diagnosis and care disparities, particularly among persons residing in the most deprived, highly segregated communities, can aid HIV prevention efforts and guide public health strategies and the equitable resource allocation needed to provide greater and better access to HIV care and other resources. In addition to addressing negative effects of segregation on affected communities, discontinuing policies that promote and sustain segregation will contribute to reducing HIV transmission and achieving health equity in the U.S. If we do not address structural racism, health disparities will continue to persist.
